# Supplementary material for: Activation of Toll-like receptors nucleates assembly of the MyDDosome signaling hub
Source: eLife. 2018 Jan 24;7:e31377. doi: 10.7554/eLife.31377 (PMC5825206; doi:10.7554/eLife.31377)
Supplement: Supplementary file 1. — The modeled TLR4/MD-2 heterotetramer exhibited increased structural drift with respect to the LPS-bound X-ray structure in the absence of ligand (apo state), as reflected in the mean RMSD values. Measurement of the surface areas buried between protein chains reveals that the largest conformational changes are evident at the primary TLR4 dimerization interfaces (which govern the stability of the higher-order heterotetrameric complex) than at the secondary TLR4 dimerization interfaces, with shifts of up to ~ 60% versus~20%, respectively. This is consistent with the observed relative motion of up to ~ 10 Å of MD2 relative to its primary TLR4 partner in the apo state, as described in the main text. [file elife-31377-supp1.docx]

| System | RMSD^a^ of TLR4/MD2 heterotetramer / nm | Area^b^ buried at 1º interface (chain A) / nm^2^ | Area^b^ buried at 1º interface (chain B) / nm^2^ | Area^b^ buried at 2º interface (chain A) / nm^2^ | Area^b^ buried at 1º interface (chain B) / nm^2^ |
| --- | --- | --- | --- | --- | --- |
| Lipid A bound | 0.22 ± 0.02 | 5.71 ± 0.26 | 5.45 ± 0.48 | 9.01 ± 0.54 | 8.02 ± 0.33 |
| Apo; replica 1 | 0.32 ± 0.03 | 3.90 ± 0.29 | 2.33 ± 0.46 | 7.42 ± 0.34 | 7.28 ± 0.39 |
| Apo; replica 2 | 0.36 ± 0.04 | 3.69 ± 0.45 | 3.99 ± 0.28 | 7.76 ± 0.35 | 8.01 ± 0.37 |
| Apo; replica 3 | 0.33 ± 0.02 | 4.23 ± 0.29 | 4.37 ± 0.37 | 7.72 ± 0.31 | 6.86 ± 0.46 |

^a^Mean root-mean-square deviation (RMSD) of all protein backbone atoms (excluding flexible termini), following least-squares fitting, measured with respect to LPS-bound X-ray structure.

^b^Mean solvent-accessible surface area (SASA) buried between each TLR4/MD2 heterodimer and either its primary (1º) or secondary (2º) TLR4 partner within the complete heterotetrameric complex. SASA was calculated using a probe radius of 0.14 nm.
